# Does Nitrogen Fertilization Affect the Secondary Structures of Gliadin Proteins in Hypoallergenic Wheat?

**DOI:** 10.3390/molecules27175684

**Published:** 2022-09-03

**Authors:** Iwona Stawoska, Jacek Waga, Aleksandra Wesełucha-Birczyńska, Michał Dziurka, Grażyna Podolska, Edyta Aleksandrowicz, Andrzej Skoczowski

**Affiliations:** 1 Institute of Biology, Pedagogical University of Krakow, Podchorążych 2, 30-084 Kraków, Poland; 2 Department of Physiology, Plant Breeding and Seed Science, University of Agriculture, Podłużna 3, 30-239 Kraków, Poland; 3 Faculty of Chemistry, Jagiellonian University, Gronostajowa 2, 30-387 Kraków, Poland; 4 The Franciszek Górski Institute of Plant Physiology, Polish Academy of Sciences, Niezapominajek 21, 30-239 Kraków, Poland; 5 Department of Cereal Crop Production, Institute of Soil Science and Plant Cultivation—State Research Institute, Czartoryskich 8, 24-100 Puławy, Poland

**Keywords:** gliadins, secondary structure, nitrogen fertilization, wheat, Raman spectroscopy, amide I

## Abstract

One of the macronutrients indispensable for plant growth and development is nitrogen (N). It is responsible for starch and storage protein (gliadins and glutenins) biosynthesis and, in consequence, influences kernels’ quality and yields. However, applying N-fertilizers increases gluten content in wheat, and it may intensify the risk of developing allergy symptoms in gluten-sensitive individuals. The purpose of our research was to analyse whether and how the elimination of N-fertilizers during the cultivation of wasko.gl− wheat (modified genotype lacking ω-gliadins) changes the secondary structures of gliadin proteins. To this aim, using the FT-Raman technique, we examined flour and gliadin protein extracts obtained from kernels of two winter wheat lines: wasko.gl+ (with a full set of gliadin proteins) and wasko.gl− (without ω-gliadin fraction) cultivated on two different N-fertilization levels—0 and 120 kg N·ha^−1^. On the basis of the obtained results, we proved that nitrogen fertilization does not have a major impact on the stability of the secondary structures of gliadin proteins for wasko.gl− wheat line with reduced allergenic properties. Furthermore, the results presented herein suggest the possibility of increasing the stability of glutenin structures as a result of the N-fertilization of wasko.gl− wheat line, which gives hope for its use in the production of wheat articles devoted to people suffering from diseases related to gluten sensitivity.

## 1. Introduction

Wheat storage proteins, comprising gliadins and glutenins (also called gluten proteins) are—from a medical point of view—important food allergens causing a range of clinical symptoms of wheat-dependent allergies [1,2,3,4,5]. One of the most important is Wheat Dependent Exercise Induced Anaphylaxis (WDEIA)—the most dangerous, life-threatening form of wheat allergy. The main source and the main reason for these unprofitable (for human health) wheat properties are the chemical properties of gliadins and glutenins. The prevalence of proline and glutamine in amino acid composition, repetitive sequences in polypeptide chains, and the presence of cysteine residues able to form intermolecular (glutenins) and intramolecular (gliadins) disulphide bonds determine their specific physicochemical properties. Both groups of gluten proteins are highly complex and polymorphic proteins. Depending on the wheat genotype, they are composed of a dozen to a hundred subunits and fractions. The range of their complexity is usually analysed by electrophoretic separation: Acid Polyacrylamide Gel Electrophoresis (A-PAGE) and Sodium Dodecyl Sulphate Acid Polyacrylamide Gel Electrophoresis (SDS-PAGE). Based on electrophoretic separations, gliadins are classified into α-, γ- and ω-groups, where: α- is the fastest and ω- the slowest migrating fraction. However, glutenins are classified based on molecular weight distribution into High Molecular Weight (HMW) and Low Molecular Weight (LMW). In previously mentioned WDEIA, a crucial role is played by a specific sub-fraction of omega gliadins—the so-called ω-5 gliadins [6]. Biosynthesis of these proteins is controlled by gene clusters localized on the short arm of 1B chromosome of hexaploid wheat [7]. Polypeptides of ω-5 fractions are composed of a long, central domain of repetitive sequences and short C and N terminal regions of specific sequences [8]. The most characteristic, repetitive sequence of ω-5 gliadins is PQQPFPQQ, in which the underlined motive makes β-turn secondary structures. WDEIA, like other food allergies, is an immunological disease. In this case, the chemical binding of gliadin molecules with specific sIgE class antigliadin antibodies plays a crucial role in pathogenesis. The hypervariable regions of IgE antibodies join short amino acid sequences called epitopes. In ω-5 gliadins, the identified epitope sequences are QQIPQQQ, QQFPQQQ, QQSPEQQ, and QQSPQQQ [9]. Other groups of α/β- and γ-gliadins also contain repetitive sequences and epitopes and are also allergenic proteins causing gastrointestinal, cutaneous, and respiratory symptoms [10].

Another important factor determining wheat proteins’ allergenic properties is the total protein content in wheat kernels, and in consequence wheat flour. This factor strongly depends on wheat genotype and the environmental conditions in which plants grew up and developed. The factors controlled by genotypes were discussed above. However, one of the most important environmental influences on total protein content is nitrogen (N) fertilization doses applied during plant development [11]. It was found that the application of N fertilizers led to a significant increase in gluten macropolymers content, total gliadin and glutenin contents, and the accumulation of individual storage protein components [12]. Studies of the response of glutenin polymerization to N fertilization in various wheat cultivars have shown that N treatment increased protein content and gluten content but also soluble and insoluble glutenin polymers and total glutenin concentration in flour [13]. The application of N fertilizers increased the synthesis and accumulation of HMW glutenin subunits. However, the content of LMW glutenin increases the most under the influence of elevating doses of N fertilizer. Nitrogen fertilization showed a more significant effect on a medium than strong-gluten wheat [14]. Therefore, wheat quality parameters may be largely modified by changing the fertilization level of nitrogen [15,16].

Total protein content and primary structures of the proteins are elementary sources of allergenic properties. However, it is highly probable that secondary protein structures are also important factors influencing allergenicity. Changing spatial conformation of protein molecules alter epitopes’ exposition to IgE antibodies [17]. Whether epitopes are exposed on the outer surface of the protein molecules or hidden in their interior, the intensity of the immunological reaction may increase or decrease [18,19]. Numerous scientific groups have undertaken various studies intending to reduce the immunogenic potential of gluten proteins by decreasing the number of highly immunoreactive protein fractions [20]. During the research carried out, we developed a range of specific wheat lines, namely wasko.gl−, lacking all of ω-gliadins belonging to both ω-5 and ω-1.2 subgroups [20]. We have observed that the elimination of ω-gliadins caused the compensation regulation effect and resulted in overexpression in other gluten protein loci—mainly that coding for some LMW glutenins and γ-gliadins. These effects were also confirmed by other authors [21]. We have evidenced a decreased IgE reactivity of the whole gliadin complex due to the elimination of ω- fractions [22]. Furthermore, we have observed and analysed differences in the gliadins’ secondary structures of wasko.gl− compared to wasko.gl+ control line [23].

More recently we focused our interests on the analysis of secondary structures in wasko.gl+ and wasko.gl− wheat lines cultivated with and without N fertilizers. The aim of these studies was to find out whether elimination of N fertilizers, during wheat cultivation, might influence gliadin protein secondary structures in ω-gliadin free wheat line (wasko.gl−) on the background of a wheat control line containing the full set of gliadins (wasko.gl+).

Nitrogen fertilization also affects the protein and gluten content in kernels. Therefore, the influence of differentiated N fertilization (0 and 120 kg N·ha^−1^) on the protein and gluten contents in two specific wheat lines (wasko.gl+ and wasko.gl−) was investigated.

## 2. Results

### 2.1. Analysis of Variance of Protein Content, Gluten Content and Gluten Index in Wheat Kernels

Protein content, gluten content and gluten index were compared in two winter wheat lines wasko.gl+ and wasko.gl− under two nitrogen fertilization doses: control, 0 kg N·ha^−1^ (N0), and 120 kg N·ha^−1^ (N120). The results of ANOVA analysis showed that statistically significant differences were found for main factors, namely wheat lines and N fertilization doses, Table 1. We found that the nitrogen dose was a primary source of variation in protein and gluten contents. The mean value of the protein content, gluten content and gluten index for winter wheat lines under N0 was relatively high, with means of 12.9%, 31.2% and 75.7, respectively, Table 2. However, under N120 the protein content and gluten content were significantly higher by 2.0% and 9.0%, respectively, and the gluten index was lower by 10.3%. The wasko.gl− line showed significantly higher protein content by 0.6% and gluten index by 44.7%. On the other hand, gluten content was lower by 6.8% compared to wasko.gl+, Table 2. There were no interactions between winter wheat lines and nitrogen fertilization doses in protein content, gluten content and gluten index.

### 2.2. Electrophoretic Measurements

**Figure 1:**
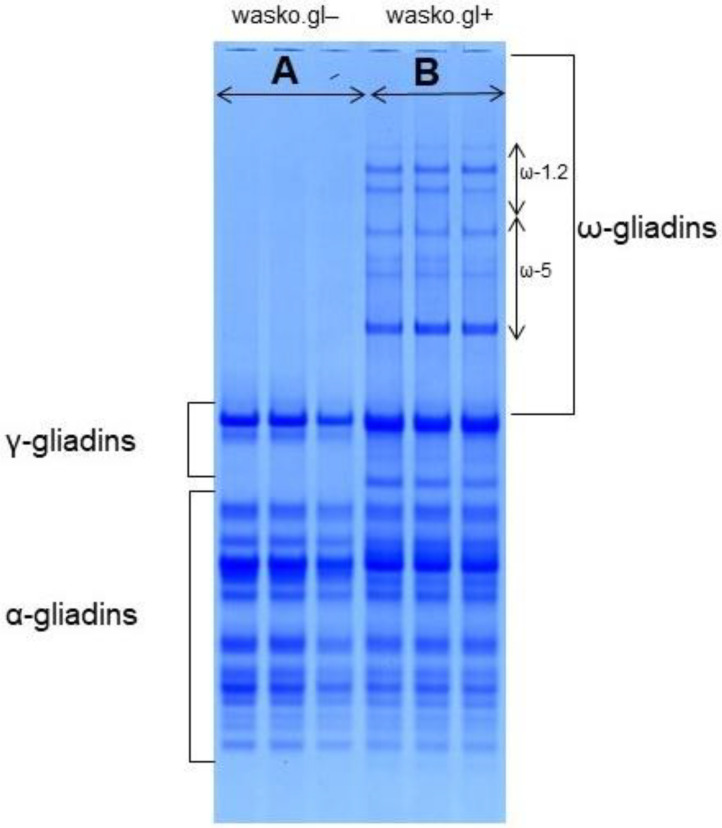
Comparison of gliadin proteins from: (**A**): wasko.gl− and (**B**): wasko.gl+ wheat genotypes separated by A-PAGE. Lack of all of ω-gliadin fractions in wasko.gl− wheat deficient line is clearly visible on three separations on the left-hand side of the electropherogram, part (**A**). In contrast, wasko.gl+ wheat control line contains all of ω-gliadin fractions belonging to the ω-1.2 and ω-5 subgroups (right hand side of the electrophoretic separation, part (**B**).

Electrophoretic patterns of gliadins extracted from wasko.gl+ and wasko.gl− wheat lines, separated by A-PAGE, display specific combinations of protein bands, Figure 1. Similar results have been already presented earlier [20]. The zones of slow migrating ω-gliadins are completely different in both genotypes. In wasko.gl+ protein bands belonging to both ω-1.2 and ω-5 subgroups are present although in wasko.gl− no protein bands in this zone were observed. Instead, the protein patterns in the lower part of the electropherogram, where fast-moving of α- and γ-gliadins occur, illustrate similar combinations of protein bands, with the exception of one γ-gliadin band clearly visible in wasko.gl+ but lacking in wasko.gl−. This results from the specificity of gliadins’ inheritance. Each gliadin locus represents a gene cluster composed of several closely linked genes, and it controls the synthesis of jointly inherited protein components—the so-called blocks. The gamma gliadin band observed in wasko.gl+ is a part of the block controlled by chromosome 1D and is composed, in addition, of three ω-1.2 bands. In consequence, silencing of the gliadin cluster localized on chromosome 1D results in the elimination of protein bands belonging to both ω and γ gliadin groups.

**Figure 2:**
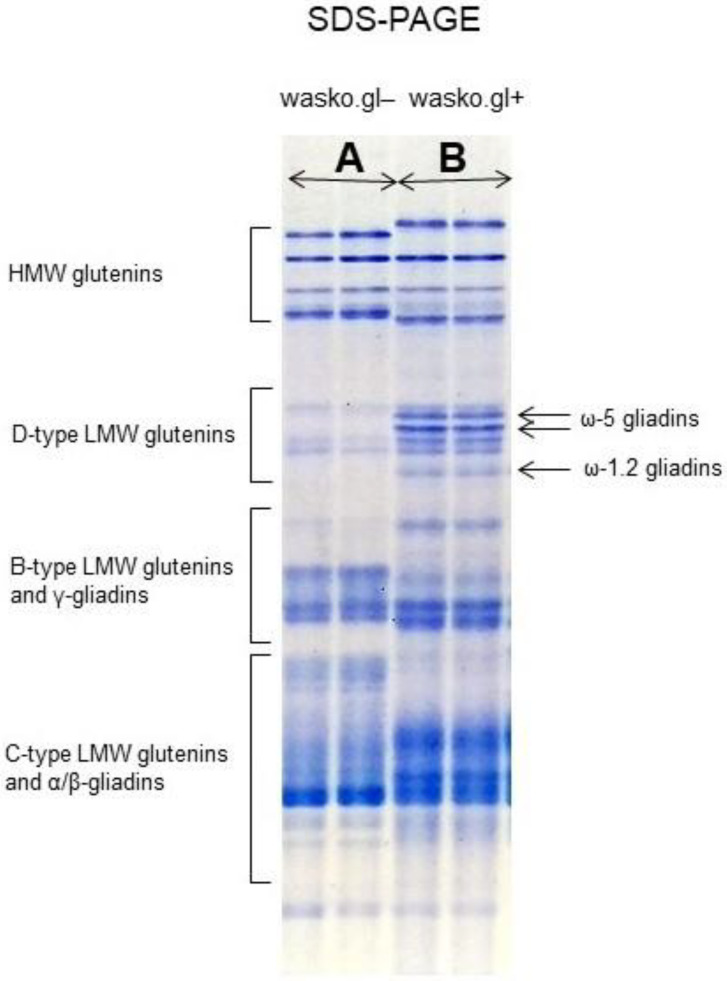
The ω-gliadins present in wasko.gl+ wheat control line, part (**B**), illustrated on the background of total proteins extracted from the endosperm of wheat kernels and separated by SDS-PAGE. ω-gliadins share the common electrophoretic zone together with D-type LMW glutenins. In wasko.gl−, ω-gliadins deficient wheat line, only D-type LMW glutenin subunits can be observed in this zone (separations on the left-hand side of the electropherogram, part (**A**). Different mobilities in HMW glutenins are due to a different genetic background of wasko.gl+ and wasko.gl−. wasko.gl− contains allele on 1B chromosome coding for 5 + 10 subunits although wasko.gl+ contains that for 2 + 12 subunits.

Elimination of ω-gliadins in wasko.gl− wheat line is also clearly visible on SDS-PAGE separation, Figure 2, see also our previously obtained results [20]. This electrophoretic method allows for the separation of total proteins of wheat kernels but is usually used (especially in genetic studies) for the identification of HMW-glutenin subunits. On the electropherogram ω-gliadins are localized in the D-zone together with D-type LMW glutenins where they form a group of five, clean-cut protein bands. In wasko.gl− wheat only three weak protein bands are observed in this zone, and they can be classified (with a high probability according to the literature data) as D-type LMW glutenin subunits [8,24].

**Figure 3:**
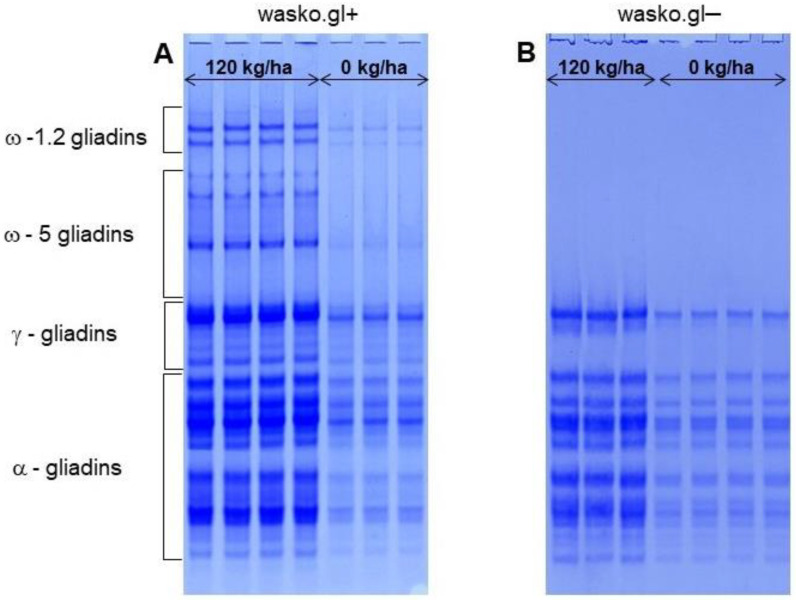
A-PAGE of gliadin proteins extracted from: (**A**): wasko.gl+ wheat control line and (**B**): wasko.gl−, ω-gliadin wheat deficient line, cultivated at the two nitrogen levels: 120 kg N·ha^−1^ (N120, left hand side) and 0 kg N·ha^−1^ (N0, right hand side of the electropherogram).

As expected, gliadins extracted from kernels developed on plots fertilized with 120 kg N·ha^−1^ (N120) and 0 kg N·ha^−1^ (N0) differed extremely regarding the staining intensity of protein bands in Coomassie brilliant blue dye solution, Figure 3. The staining intensity of all gliadin bands strongly decreased when comparing the samples from fertilized plots (120 kg N·ha^−1^) to that from no fertilized ones (0 kg N·ha^−1^). However, the strongest effect (close to disappearance) was observed for wasko.gl+ among ω-gliadins compared to the samples collected from the plots fertilized by 120 kg N·ha^−1^. Only traces of proteins were observed among α-gliadins, although β and γ gliadin bands were visualized a little bit stronger.

### 2.3. FT-Raman Measurements and Curve Fitting Analysis

**Figure 4:**
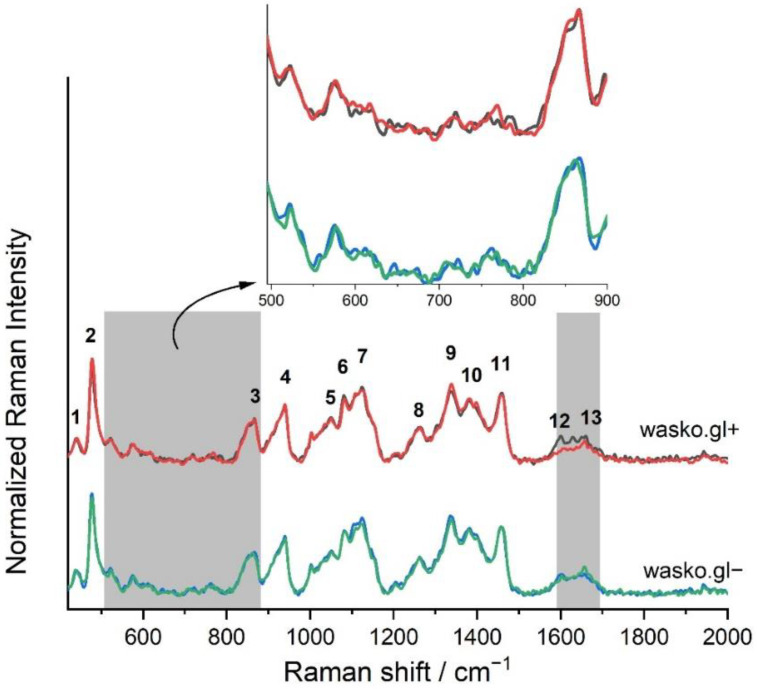
The normalized FT-Raman spectra of flour of two wheat lines lacking (N0) or N-fertilizers treated (N120): wasko.gl+ (red (N120) and black (N0) solid lines) and wasko.gl− (blue (N0) and green(N120) solid lines). Numbers 1–13 indicate bands typical for the main chemical components in kernels and they are characterized in Table S1 in Supplementary Materials. Inset: the spectroscopic region, 500–900 cm^−1^, typical for amino acids and SS vibrations; N ≥ 3, SD < 5%.

The used FT-Raman spectroscopy method allowed for the characterization of flour obtained from kernels of both tested lines, namely wasko.gl+ and wasko.gl−, lacking (N0) or treated (N120) with nitrogen fertilizers. The vibrations deriving from typical biochemical compounds, that are the main components of wheat flour, are pointed in Figure 4 and characterized in Table S1 (in Supplementary Materials).

The main constituent of wheat seeds is starch which builds up to 80% of their total mass. The intensive Raman bands at 480 and 938 cm^−1^ (peaks 2 and 4 respectively), known as marker bands for starch, are clearly visible in the obtained spectra. According to Kizil et al. and Almeida et al. the changes in the position and intensity of the latter one, which is mainly due to α-1,4 glycosidic linkage, allow for the study of the content of amylose and amylopectin in samples [25,26]. The regions 800–1200 and 1200–1500 cm^−1^ are rich in vibrations representative of carbohydrates, and they are related to C-C, C-O stretching, C-O-H deformation modes owing to glycosidic bonds and to C-H, C-O-H deformation, C-C-H coupling vibrations, respectively. In these regions, change in the intensities of selected peaks are visible, Figure 4, which suggests that nitrogen fertilization influences the biochemical composition of both wheat lines, especially the carbohydrate content in wheat. This fact is in line with the already published results [27,28,29]. However, visible changes in Raman spectra obtained for studied wheat lines wasko.gl− and wasko.gl+, treated with various doses of N fertilizers, are observed in the range of 500–900 cm^−1^. This is characteristic for amino acid vibrations, and also in the range of amide I, namely 1590–1710 cm^−1^, which is sensitive for secondary structural changes of proteins, Figure 4, grey shapes, and the inset. The first range (500–900 cm^−1^) is representative of vibrations coming from selected amino acids and their characteristics allow for the study of the local conformation of amino acid residues in the protein chain. It should be noted that these variations may occur under interactions with the environment, denaturation, structural changes, as well as the formation of protein polymer structures.

Folded conformations of many proteins are stabilized by disulphide bonds which also play a key role in determining the structure of wheat gluten proteins. Concerning the distribution and localization of Cys residues in the amino acid sequences of wheat prolamines, the classification into sulphur-rich, sulphur-poor, and high molecular weight (HMW) groups is used. The first group comprise the monomeric α-type gliadins (precisely α- and β-gliadins), monomeric γ-type gliadins and low molecular weight (LMW) subunits of glutenins (B- and C-types). These types of proteins possess similar structures and conserved cysteines responsible for intra-chain disulphide-bonds formation that stabilize the folded conformation. However, B and C-types of LMW glutenins go to the S-rich group of prolamines, ω-gliadins (contain no cysteine residues) and the D-type of LMW glutenins, also called “aggregated gliadin” [30], that are “mutant”-like forms of ω-gliadins with a single Cys residue that allows crosslinking (by inter-chain disulphide bonds) into the glutenin polymers, belongs to the S-poor prolamines [31,32,33]. The last groups are polymeric HMW prolamines that are related structurally and evolutionary to both S-poor and S-rich prolamines [34] and for these, inter-chain disulphide bonds are formed leading to the formation of insoluble aggregates. Previously obtained results have shown that there are three parts of gluten proteins that are responsible for gluten allergenicity, namely a short amino acid sequence that probably acts as an antibody-binding epitope in immunological reactions, β-turns responsible for a specific conformation of biomacromolecules and SS bonds with their stabilizing activity [35]. Using Raman techniques it is possible to characterize the conformation of disulphide bonds on the basis of bands localized in the range 490–550 cm^−1^ [36]. The content of selected conformation (in percentage) of SS bridges calculated based on the mathematical decomposition of the 490–550 cm^−1^ region is shown in Table 3 and in Figure S1 in the Supplementary Materials. For N120 wasko.gl− wheat line (without ω-gliadins fractions), the SS_gauche-gauche-gauche_ (SS_g-g-g_) and SS_trans-gauche-trans_ (SS_t-g-t_) content increased, and SS_trans-gauche-gauche_ (SS_t-g-g_) decreased compared to N0. Changes in the conformation of SS bridges for wasko.gl+ line (with the full set of gliadins) were also observed. After nitrogen fertilization, N120, the highest content was observed for SS_t-g-t_ structures and no SS_g-g-g_ forms were found compared to N0.

In the range 630–730 cm^−1^, Figure 4 inset, stretching vibrations from Met and Cys molecules are also visible. However, due to the low signal intensity, it is difficult to discuss the conformation of these amino acid residues present in flour from tested wheat lines. It is worth adding that bands at approximately 650 cm^−1^, responsible for C–S stretching vibration in gauche conformation [36], are evident only for N0 samples of wasko.gl+ and wasko.gl− lines; however bands at 720 cm^−1^ are detected for both wheat lines regardless of nitrogen fertilization.

Besides covalent disulphide bonds, also hydrogen bonds, the non-covalent ones play an important role in protein folding. In this type of interaction, tyrosine residues play an important role. The tyrosine moiety (Tyr) possesses bands at 855 and 835 cm^−1^ in Raman spectra and analysing the intensity ratio I(855 cm^−1^)/I(835 cm^−1^) it is possible to discuss the interaction between OH of Tyr moiety and the vicinity of it. Siamwiza et al. proved that the doublet bands are due to Fermi resonance between the ring-breathing vibration and the overtone of the out-of-plane ring-bending vibration [37]. According to the literature data, if the tyrosine doublet ratio belongs to the range 0.9–1.43, tyrosines can function as donors or the acceptors of protons and below the 0.9 value, the phenolic group is buried and is acting rather like a donor of strong H-bonds. Conversely, the exposition of this amino acid on the surface of the protein leads to an increase in the disused ratio [37,38]. In the presented results for both tested wheat lines, regardless of nitrogen fertilization, the Tyr doublet was higher than 1.5, which is in line with our previous observation [23] and also with the results obtained by others researchers [39,40]. However, for wasko.gl+ we observed no influence of nitrogen fertilization on the conformational Tyr modification, for wasko.gl− an increase in the value of the intensity ratio as the response of fertilization was observed.

The vibration typical for tryptophan (Trp) residues at 1360 cm^−1^ is usually an indicator of the exposition of the indol ring into the environment. Based on the obtained spectra, we can suggest that the indol ring is exposed to the environment in both wheat lines regardless of N-fertilization. Also, the vibration at 760 cm^−1^, characteristic of the strength of H-bonds formed between Trp and polypeptide chain in the close vicinity shows that this amino acid is rather exposed outside the protein globule.

A second spectroscopic region in which visible changes, as a consequence of a nitrogen fertilization of wheat lines wasko.gl+ and wasko.gl−, were detected is connected with the amide I band (1590–1710 cm^−1^), Figure 4, grey selection. The detailed analysis of these bands allowed for the identification of secondary structures of proteins that build investigated samples. Since in wheat flour there are not only gluten proteins but also albumins, globulins, and others, we decided to isolate the gliadins from the whole bulk and analyse changes in the secondary structures of this group of proteins. We choose gliadin proteins for further analysis because of the structural differences between wasko.gl+ and wasko.gl− lines are connected mainly with the presence or lack of the ω-gliadin fraction, Figure 1.

**Figure 5:**
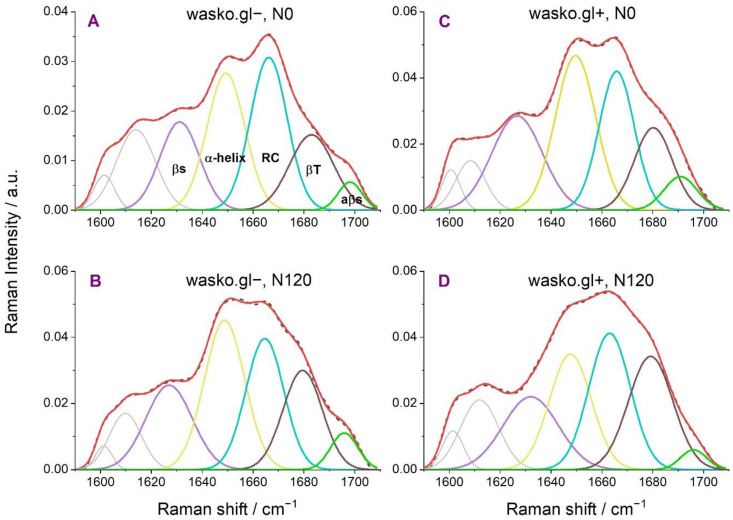
Curve fitting of the Raman spectrum in the range of amide I obtained for gliadin proteins isolated from flour of the wheat lines wasko.gl− and wasko.gl+. Spectra (**A**,**B**) show results obtained from flour of wasko.gl− wheat kernels under minimal (N0) and maximal (N120) nitrogen fertilization, respectively. Spectra (**C**,**D**) show results obtained from flour of wasko.gl+ wheat kernels under minimal (N0) and maximal (N120) nitrogen fertilization, respectively. The experimental spectra are represented by dashed black lines and the calculated ones are represented by solid red lines. The calculated profiles in the panels were determined as the sums of the individual curve-fitted components, namely, β-sheet (1627–33 cm^−^^1^—violet), α-helical (1648–50 cm^−^^1^—yellow), random coil (1663–1666 cm^−^^1^—blue), β-turn (1680–83 cm^−^^1^—brown), and aβ-sheet (1691–98 cm^−^^1^—green) structures.

Based on the obtained results, we detected only a slight effect of nitrogen fertilization on gliadins in wasko.gl− line (without ω–fractions), Figure 5A,B and Table 4. Almost no differences in the percentage of secondary structural content between N0 and N120 were found. Additionally, only a slight decrease in the random coil, RC, or undefined structures for wasko.gl− at the expense of β and α structures under N120 was found. What draws attention, however, is the noticeable effect of nitrogen fertilization on the secondary structures in gliadins wasko.gl+ line (with the full set of gliadins), which was estimated, Figure 5C,D and Table 4. For N120 an increase of β–turn and in, to a lesser extent, RC/undefined structures were detected at the expense of α–helix and β–sheets. Furthermore, an additional two peaks at 1602 cm^−1^ and 1609–1614 cm^−1^ were identified as aromatic side chain vibrations typical for phenylalanine (Phe), tryptophan (Trp) and tyrosine (Tyr) residues [41,42].

### 2.4. HPLC Analysis

RP-HPLC analyses revealed that N treatment was reflected in gliadin fractions’ quantitative and qualitative composition. In all, 72 gliadin bands of ω, α/β, and γ group were monitored. Objects with lowered gluten content (wasko.gl−) reacted on N120 dose, reducing 19 gliadin bands, including 10 of ω (in 2–43% range), 3 of α/β (of 41, 44, and 63%), and 6 of γ (in 4–45% range) fractions. However, an abundance of 44 bands were altered (19 of ω (1–77% range), 12 of α/β (7–94% range), and 13 of γ (8–46% range)) at the same time.

The N treatment resulted in a 19% increase in total gliadin content.

**Figure 6:**
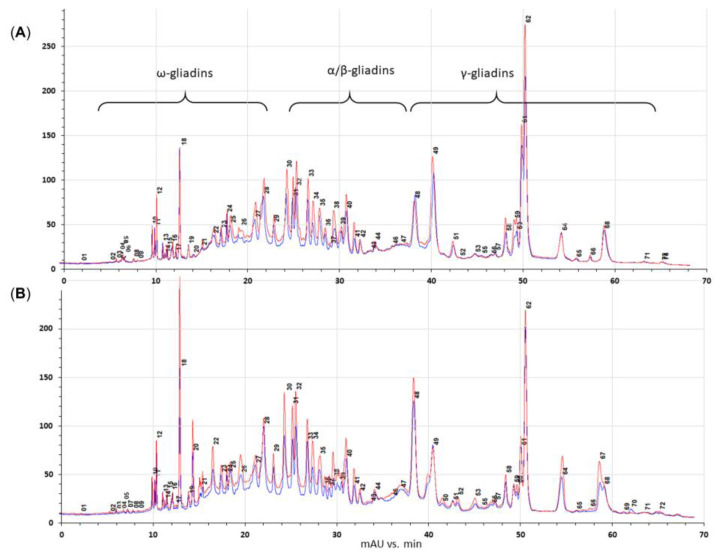
Comparison of reverse-phase high-performance liquid chromatography (RP-HPLC) chromatograms of gliadin factions from wasko.gl− ((**A**), upper traces) and wasko.gl+ ((**B**), lower traces) wheat genotypes, with different N treatments (blue—N0 and red—N120).

The control line (wasko.gl+) comparably reacted to N fertilization, as 13 bands were lowered (6 of ω (1–18% range), 1 of α/β (28%), 7 of γ (in 2–200% range)), besides 1 completely decayed ω and 2 γ bands. At the same time, fertilization altered 54 bands (24 ω (3–100%), 14 α/β (14–149%), 16 γ (3–231%)), including the appearance of 2 gamma bands. In total, fertilization increased gliadin accumulation by 25%. The numerical details are given in Supplementary Table S2. The representative chromatogram of wasko.gl− and wasko.gl+ and their N fertilization variants are presented in Figure 6.

**Figure 7:**
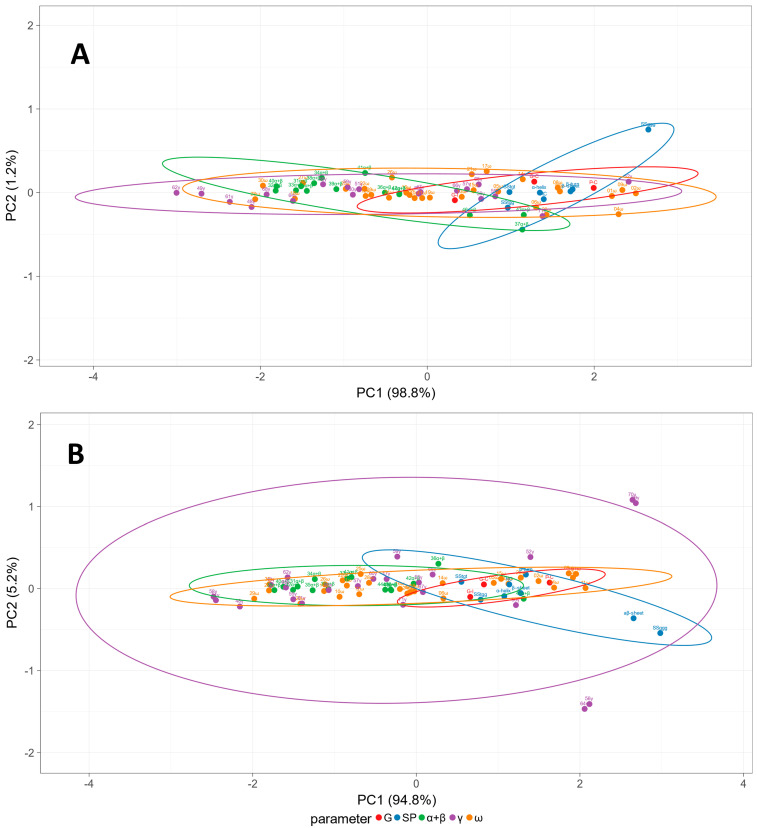
Principal component analysis (PCA) plot of measured parameters: gluten content, gluten index, and protein content (G); secondary structure parameters estimated from Raman spectra (SP); and proportions of gliadin fractions (α+β, γ, ω). Prediction ellipses mark an area where a new observation from the same group will fall with a probability of 0.95. The upper panel (**A**) presents results for wasko.gl− whereas lower panel (**B**) presents data for wsko.gl+.

The relationship between parameters such as gliadin profile (ω, α+β, and γ relative abundance), secondary structure elements derived from Raman data (SP, presented in Table 3 and Table 4)), gluten index, protein, and gluten content (G, based on Table 2) was presented in Figure 7 after conducting principal component analysis (PCA). This approach seemed justified, taking into account a relatively significant number of parameters. However, for both wheat genotypes (wasko.gl− and wasko.gl+), the analysis revealed two principal components (PC), explaining 98.8% (PC1) and 1.2% (PC2) and 94.8% (PC1) and 5.2% (PC2) of total variability, respectively for wasko.gl− and wasko.gl+ genotypes. For wasko.gl− most of the parameters weakly correlated with PC2. Only SS_ggg_ conformation of sulphur bridges correlated more visibly with that component. On the other hand, most parameters were more strongly correlated with PC1. Parameters describing the secondary structure, protein, and gluten content and lighter, less hydrophobic, ω and γ gliadin fractions correlated strongly positive with PC1. At the same time, most of the other gliadins correlated negatively with PC1. The PCA analysis in wasko.gl+ gave a different picture. A stronger correlation between measured parameters and PC2 could be seen. The PC2 relation with Raman spectra-derived secondary structures was inverted compared to wasko.gl−. Interestingly, less polar γ gliadin fractions (as bands 56 and 64) correlated positively, and more polar γ gliadin fractions (as bands 69 and 70) correlated negatively with PC2. The correlation between most other parameters and PC1 was like the situation described for wasko.gl−.

**Figure 8:**
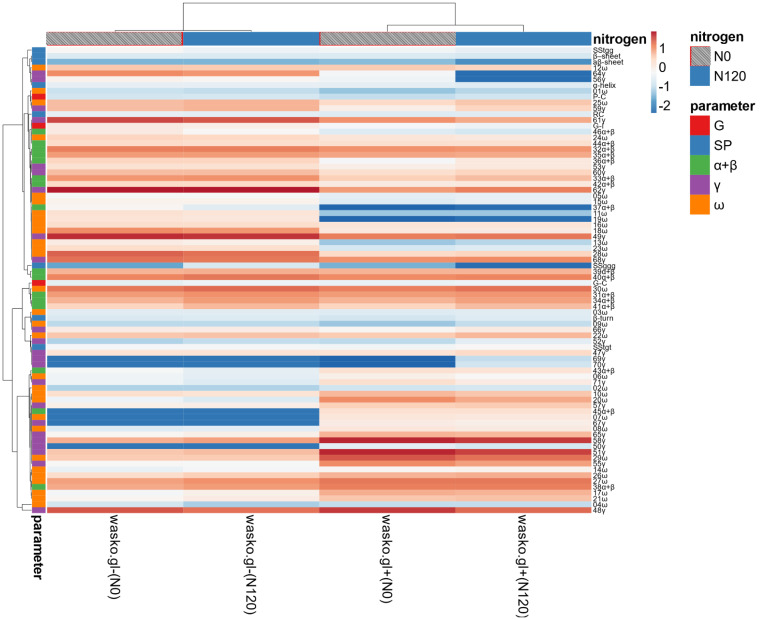
Heat map of analysed parameters: gluten content and index, and protein content (G); secondary structure parameters estimated from Raman spectra (SP); and proportions of gliadin fractions (α+β, γ, ω). Original values are ln(x)-transformed. Unit variance scaling is applied to columns. Both rows and columns are clustered using correlation distance and average linkage. Coloring intensity represents the relative value of a particular parameter. Red shades represent accumulation, whereas blue shades represent parameter decrease.

To give a more complementary picture of relations between different variables, the data matrix of estimated parameters for both wheat forms in different N fertilization regimes is visualized in Figure 8. Numerical differences are expressed by colouring and its warmth. It is visible that both wheat forms are separated regardless of N fertilization regimes. N fertilization was much less pronounced than effects connected with gluten allergenicity reduction between wasko.gl− and wasko.gl+. Most Raman spectra-derived parameters describing protein secondary structures clustered with low polar α+β and γ gliadin fractions and protein content and gluten index. The most pronounced effect of N fertilization could be noticed for a fraction of γ gliadins, which tended to be reduced after N120 fertilization, regardless of wasko.gl− or wasko.gl+ properties.

## 3. Discussion

The genotype, growing condition, as well as their interaction, play a significant role in determining the grain quality of wheat. In particular, nitrogen fertilization is one of the agronomic practices to achieve high protein and gluten content [43,44]. The nitrogen supply of wheat plants throughout the growing season is very important for plant development, especially for all leaves, and consequently affects the protein and gluten content. This is due to the fact that N accumulated in the vegetative organs before anthesis is redistributed into the grain accounts for more than 75% of the total N in final dry wheat grain, although N absorbed and directly transported to the grain after anthesis accounts for less than 25%. Thus, the pre-anthesis accumulated N is crucial for the accumulation of storage protein in wheat grain, and to a large extent, determines the processing quality of wheat flour [45,46,47].

In the presented studies we analysed the protein and gluten content and gluten index in winter wheat lines under control with no nitrogen (0 kg N·ha^−1^, N0), and high nitrogen (120 kg N·ha^−1^, N120) applied. Our experiment was conducted on a field where the N level of the soil equalled 17.2 mg·kg^−1^. The nitrogen fertilization was done in a dose of 120 kg N·ha^−1^ in two growth phases: the first dose after the resumption of spring vegetation (60 kg N·ha^−1^) and the second when plants had reached the stem elongation stage. Firstly, we analysed the protein and gluten content and gluten index in winter wheat lines under control (no nitrogen applied) and high nitrogen (120 kg N·ha^−1^). Statistically significant differences were found for the main factors (winter wheat lines, N level) which are in accordance with the results of other authors [48,49,50,51]. We found that the nitrogen dose was a primary source of protein and gluten content variation. The protein content equalled 12.9% (N0) and 14.9% (N120), Table 2. The lower protein content in wheat grain with N0 is due to the fact that fertilizer application had a significant effect on the leaf metabolome profile. N-deficient plants accumulate less total N, S, and free amino acids compared with no N-deficient plants. Also, the senescence in N-deficient plants occurred earlier than in the higher N treatment. Because a high N supply increases cytokinins’ concentration, preventing leaf senescence and proteolysis, N-deficient plants contained less N to remobilize [46,47]. On the other hand after N0 treatment, the protein content and gluten content were high (12.9% and 31%, respectively). Generally, the application of high-N fertilizer (120 kg N·ha^−1^) compared to N0 increases the protein content only by 2%, and by 9% in gluten content. The slight increase in protein content is due to the fact that during grain filling, when soil N supply is ample, the N uptake machinery by the root is repressed due to the high amino acids concentration in the tissues, resulting in low N uptake despite its availability, since a great proportion of the fertilizer remains in the soil [47].

The differences in the amount of gluten under N fertilizer may result from the differential expression of genes in the cultivars. Yu, et al. [52] suggested that nitrogen treatment increased the storage protein content, endosperm protein body quantity, and partial processing quality by altering the expression levels of certain genes involved in protein biosynthesis pathways and storage protein expression at the proteomics level. The positive effect of high doses of nitrogen on the amount of gluten had also been reported by other researchers [13,15,16].

Gluten, made of glutenin and gliadin proteins, is responsible for forming a viscoelastic network in the dough. It is already known that the addition of N fertilizers increases the content of glutenins, mainly high molecular-weight glutenins, allowing for a more significant amount of SS bond formation and, as a result, improvement in the bread-making quality [53]. High and low molecular subunits of glutenins were connected via covalent SS bonds. However, glutenins and gliadins interacted mainly via hydrogen or non-covalent bonds [24,54,55].

To find an explanation for the structural changes of gluten proteins in tested wheat lines, namely wasko.gl+ (with the full set of gliadin proteins) and wasko.gl− (without ω-gliadin fractions) under nitrogen fertilization, the FT-Raman spectroscopy method was used. Firstly, Raman spectra of wheat flour of wasko.gl+ and wasko.gl− obtained from kernels of non-treated (N0; 0 kg N·ha^−1^) and treated (N120; 120 kg N·ha^−1^) with nitrogen fertilizers were done. It was found that changes in disulphide bridges’ conformations were more pronounced after nitrogen fertilization for wasko.gl−. For N120 samples, there was a significant increase in SS_ggg_ (13%) and a relatively slight increase in SS_tgt_ (3%) forms, and a decrease in SS_tgg_ structures (16%) compared to N0, Table 1. For N120 of wasko.gl+ samples, an increase in SS_tgt_ (17%) and a decrease in both SS_ggg_ (5%) and SS_tgg_ (12%) bridges were observed compared to N0. Based on the obtained results, it can be concluded that for the wasko.gl−, which was devoid of ω-gliadin fraction, nitrogen fertilization led to the formation of SS_ggg_ disulphide bridges, which are typical of the most stable protein structures formed in place of SS_tgt_ conformation with relatively low stability. The opposite effect was observed for flour of kernels of wasko.gl+ line. For the control line, an increase in less stable forms of SS bonds, namely SS_tgt_ and a decrease in stable structures, namely SS_ggg_ and SS_tgg_, were observed after nitrogen fertilization. To conclude, N fertilization affects the gluten structure of both tested wheat lines. However, the presence or absence of ω-gliadin forms in protein complexes leads to a completely different effect on the conformation of disulphide bridges. Consequently, this may influence the protein stability and/or possible aggregation processes.

The conformational changes of SS bridges in gluten were previously examined by Nawrocka et al. however, the experiments were associated with various fibre additives to gluten samples [39,56]. The authors presented a decrease in the most stable SS_ggg_ bridges and an increase in SS_tgg_ and SS_tgt_ structures after adding dietary fibre to pure gluten samples. Our previous studies proved that the elimination of ω-gliadin fractions from protein clusters could stimulate conformational changes in protein structures [23]. We found that for wasko.gl+, with a full set of gliadin protein, the SS_ggg_ bonds conformation, typical for more stable forms, was detected in a higher content in the endosperm compared to wasko.gl−. However, based on the presented results, it seems possible to influence the disulphide bridges’ conformations with nitrogen fertilization. Since SS bonds are mainly found in glutenin structures and play a crucial role in protein folding and the formation of higher-ordered forms, conformational changes in this group of gluten proteins may lead to changes in the strength and elasticity of bread dough. They can also affect the allergenic properties of gluten proteins.

In gluten proteins, tyrosine molecules are also detected as a doublet of peaks at 855 and 835 cm^−1^ in Raman spectra. The ratio of intensities of these peaks allows us to discuss the contribution of Tyr moieties to intra- and intermolecular interactions. The value of the ratio I(855 cm^−1^)/I(835 cm^−1^) obtained for both N0 samples of flour from the wheat of wasko.gl+ and wasko.gl− equals 1.86. This result suggests a possible partial exposition of Tyr molecules on the surface of the protein, which is in line with previously obtained results [23]. However, for wasko.gl+ no changes of the tyrosine doublet under nitrogen fertilization were observed, for wasko.gl− the positive shift from 1.86 for N0 to 2.13 for N120 was detected. The obtained value suggests an increase in the role of phenolic oxygen as the acceptor atom in an H-bond formation.

Instead, the oscillations characteristic of Trp (at 1360 cm^−1^ and 760 cm^−1^) suggest that these amino acid residues are exposed on the surface of the protein complex of wasko.gl + and wasko.gl- regardless of whether N fertilization was applied or not.

Obtained findings for wheat flour correlate well with results achieved by FT-Raman spectroscopy for gliadin protein fractions isolated from wheat flour of both lines, namely wasko.gl+ and wasko.gl−. Using mathematical decomposition of the amide I band, the presence of ordered secondary structures of gliadin proteins in the wasko.gl− line was confirmed, regardless of nitrogen fertilizers. Thus, using nitrogen fertilizers in the case of wheat with reduced allergenic properties (devoid of the ω-gliadin fraction) does not adversely affect the secondary structural changes of gliadin proteins. At the same time, the conformational stability of covalent disulphide bonds presents mainly in glutenin structures increases after the application of nitrogen fertilizers for wasko.gl− wheat line. Simultaneously, it was noticed that the use of N fertilizers for wasko.gl+ wheat line caused both an increase in less stable disulphide bonds present in gluten proteins, as well as an increase in random and/or disordered structures and β-turns at the expense of the decreasing number of α-helix and β-sheet forms for gliadins in the wasko.gl+ line.

To conclude, using nitrogen fertilizers for wasko.gl− wheat line, which is devoid of allergenic ω-fraction, it is possible to increase the content of proteins without significant changes in secondary structures of gliadin proteins together with an increase in stability of disulphide bridges in gluten proteins. However, for wasko.gl+ wheat lines decomposition of amide I band registered for gliadin proteins allows for detection of secondary structural modifications under nitrogen fertilization (N120 samples). The changes attesting to an increase in the formation of RC and β-turs structures may influence the protein stability and, in consequence, lead to aggregation of gluten (especially gliadin) proteins. These observations also agree with Trp exposition on the protein surface and its possible involvement in hydrogen bond formation. One should also not forget about the fact that for the wasko.gl+ line, changes in the conformation of SS bonds were observed after nitrogen fertilization, indicating the formation of less stable structures. Noteworthy is the fact that the band at 1609–1614 cm^−1^ classified here as aromatic amino acid (Phe, Tyr, Trp) side chain vibrations, is also defined in literature as the one that characterizes aggregated structures of β-sheets with intermolecular hydrogen bonds [57,58]. For gliadins obtained from wasko.gl− wheat line after fertilization (N120) a decrease in the percentage content of the band compared to N0 was observed. The opposite effect for wasko.gl+ wheat gliadin samples were found. Thus, it is likely that not only aromatic amino acid side chain vibration but also an amount of β-sheets aggregated structures are part of this band.

Based on the obtained results, it is suggested that nitrogen fertilization does not have a major impact on the stability of the secondary structures of gliadin proteins for wasko.gl− wheat line with reduced allergenic properties. In addition, the results presented in the study suggest the possibility of increasing the stability of glutenin structures due to nitrogen fertilization of wasko.gl− wheat lines, which gives hope for its use in the production of wheat products dedicated to people suffering from diseases related to gluten sensitivity. However, these hypotheses need additional investigations concerning glutenin polymers.

## 4. Materials and Methods

### 4.1. Plant Materials

For all experiments two winter wheat (*Triticum aestivum* L.) lines were used: wasko.gl− and wasko.gl+ [20]. Field trials were conducted during 2019–2020 and 2020–2021 growing seasons at the Experimental Station in Osiny (51°28′45 N 22°03′16 E), Institute of Soil Science and Plant Cultivation—State Research Institute (IUNG-PIB) Pulawy, Poland.

The field experiment was designed in 3 replications (plot size 2.5 × 10.0 m), each replication was grown in a separate block, where the field plots were randomized. The soil was pseudopodzolic, which is a typical soil type for the region. pH was 6.2; 22.1 P_2_O_5_, 28.6—K_2_O, Mg 6.5 mg·kg^−1^ of soil. Soil samples for the measurement of mineral N (N-NO_3_^–^ + N-NH_4_^+^) were collected from the 0–30, 30–60 and 60–90 cm depth in early spring prior to fertilizer application. The test showed the N level in the soil equalled 17.2 mg·kg^−1^ of soil. Winter rape was the fore crop. Winter wheat was sown at the seed rate of 3.5 million·ha^−1^. The sowing terms were: 4 October 2019 and 9 October 2020.

In every year, complex mineral fertilizers (P_2_O_5_ 60 kg·ha^−1^, K_2_O 90 kg·ha^−1^) were applied before sowing in the whole experimental field. In this experiment, we focused on studying two factors: wheat line (wasko.gl+ and wasko.gl−) and nitrogen fertilization dose. The two levels of nitrogen were used: no nitrogen (control, N0) and 120 kg N·ha^−1^ (N120) in two doses of N 60 + 60 kg N·ha^−1^ were applied. The first dose after resumption of spring vegetation (60 kg N·ha^−1^) and the second when plants reached the stem elongation stage. Weeds were controlled by the recommended herbicides in the autumn and spring. Seed treatment, plant growth regulators, fungicides as well as insecticides were applied only at the recommended rates and time. Harvest term was in the full maturity stage (31 July 2020 and 30 July 2021).

### 4.2. Kernels Quality Characteristics

Protein content (ISO 20483:2013), gluten content (ISO 21415-2:2015) and gluten index (ISO 21415-2:2015) were determined to assess the baking quality parameters of wheat flour. The content of nitrogen (N) was evaluated using CFA with spectrophotometric detection methods (PB 033. 24.02.2020). N content calculation for a crude protein was based on the multiplication of the N result by the conventional factor 5.7 (ISO 20483). Wet gluten content (GC) and quality, assessing the gluten index (GI), were analysed using Glutomatic System (Perten Instruments, Hägersten, Sweden) in accordance with the standardized Perten method (ICC 155).

### 4.3. Wheat Sample Preparation for HPLC and FT-Raman Measurements

Samples of whole kernels were preliminarily prepared by grinding in a mortar and next used for HPLC (Agilent Technologies, Woldbron, Germany) and electrophoresis analysis (Hoefer, Holliston, MA USA) as well as FT-Raman analysis of biochemical components (Thermo Scientific Nicolet NXR 9650 FT-Raman spectrometer, Waltham, MA, USA) measurement.

### 4.4. Gliadins and Total Protein Extraction

Gliadins’ extraction prior to FT-Raman measurements was done using 70% EtOH after removing water/salt soluble albumins and globulins. To this aim 30 mg of wasko.gl+ and wasko.gl- flour samples were mixed with 300 μL 0.15 mol·dm^−3^ NaCl for 2 h with gentle shaking at room temperature, and centrifuged for 10 min., 12,000 rpm. After the first step of extraction supernatants containing albumins and globulins were removed and 300 μL of 70% EtOH was added to each pellet and shaken overnight at room temperature. Then, the obtained extracts were centrifuged again (10 min., 12,000 rpm). The supernatants were collected and immediately used for further analysis.

Total proteins were extracted from flour samples (30 mg) using 300 μL of extraction solution composed of 6M urea, 1% SDS and 2% mercaptoethanol.

### 4.5. Gel Electrophoresis

Gliadins and total proteins were analysed by Acid Polyacrylamide Gel Electrophoresis (A-PAGE) and Sodium Dodecyl Sulphate Polyacrylamide Gel Electrophoresis (SDS-PAGE) as described earlier [20]. Shortly, acidic gel for gliadins was composed of total monomers in concentration T = 8% (*w*/*v*) and crosslinker (methylene bisacrylamide) concentration C = 0.29% (*w*/*v*). Separations were carried on in aluminium lactate buffer (Ph = 3.1) at constant voltage (U = 500 V). for about three hours. Total proteins were separated in the basic gel of total monomers concentration T = 10% and methylene bisacrylamide concentration C = 1.5%. Electrophoresis was run in Tris-HCl buffer, pH = 8.9 at constant current I = 90 mA for about four hours. Both protein groups were separated in VWR electrophoretic chambers.

### 4.6. Reverse-Phase High-Performance Liquid Chromatography (RP-HPLC) of Gliadin Fraction

Changes in gliadins’ proportions were estimated using RP-HPLC [20,59]. Analyses were done exactly as published earlier [23]. Briefly, milled material (100 mg) was extracted in 70% ethanol. Supernatant after centrifugation was analysed by RP-HPLC. Agilent Infinity 1260 (Agilent Technologies, Waldbronn, Germany) with a DAD detector was used for analyses. Gliadins were separated on an AdvanceBio RP-mAb SB-C8 (SB-C8) 4.6 × 150 mm; 3.5 µm particles size (Agilent Technologies, Santa Clara, CA, USA) maintained at 60 °C. A gradient of H_2_O and acetonitrile, with 0.1% trifluoroacetic acid (TFA) in both phases was used. Absorbance was recorded at 210 nm.

### 4.7. Raman Spectroscopy Measurements and Curve Fitting Analysi

FT-Raman spectra were recorded with a Thermo Scientific Nicolet NXR 9650 FT-Raman spectrometer (Madison, WI USA) equipped with a Micro-Stage Microscope and the InGaAs (indium gallium arsenide) detector. The samples were excited with a 1064 nm line of the Nd:YAG^3+^ laser. FT-Raman measurements were performed for (i) flour obtained from kernels of wasko.gl+ and wasko.gl− lines, and for (ii) the gliadins extracted (in 70% ethanol) from flour obtained from the above-mentioned wheat lines. Both wheat lines were (N120) or were not (N0) nitrogen fertilized. The spectra were collected in the range of 4000–300 cm^−^^1^, accumulated from 2000 scans and measured with the laser power of 0.4 W for flour or 0.8 W for alcoholic solutions. Analyses were done according to a previously published procedure [23]. Concisely, spectra obtained for flour followed the baseline correction and normalization at 1460 cm^−1^ were analysed in the range of 2000–300 cm^−^^1^. The OriginPro 2020 software package for Windows was used for mathematical analysis. Determination of the percentage distribution of SS bridges’ conformation of gluten proteins was obtained based on the decomposition of the bands in a range of 500–550 cm^−^^1^. Determination of the secondary structures of gliadin proteins extracted from wheat kernels of wasko.gl+ and wasko.gl− lines were achieved on a decomposition of the amide I (1590–1710 cm^−^^1^) band registered for liquid samples. For the decompositions calculations, the PeakFit 4.12 (Systat Software, Inc., Palo Alto, CA USA) program was used according to the modified procedure described earlier [60,61]. The analysis of the conformations of SS bridges, as well as components of the amide I band, was done according to the already recognized criteria [36,38,41,42,62,63,64,65,66,67]. Regarding disulphide bridges’ conformations and the secondary structures of gliadins estimated from amide I decomposition, the correlation coefficient was not lower than 0.997 and 0.999, respectively.

### 4.8. Statistical Analysis

Differences in protein content, gluten content and gluten index between the nitrogen fertilization doses and winter wheat lines were estimated using a two-way analysis of variance (ANOVA), and homogeneous groups were determined by Tukey’s test at a significance level of 0.05. The calculations were performed using Statistica 13.1 software.

PCA and a heat map were done with the use of an online tool (http://biit.cs.ut.ee/clustvis/ accessed on 29 April 2022) according to Metsalu and Vilo [68]. Singular value decomposition (SVD) with imputation was used to calculate principal components. Original values were ln(x)-transformed. Unit variance scaling was applied to rows; singular value decomposition (SVD) with imputation was used to calculate principal components.

## Figures and Tables

**Table 1:** Basic statistical parameters of the variability of the examined traits.

Trials	Protein Content (%)		Gluten Content (%)		Gluten Index	
	F	*p*	F	*p*	F	*p*
Line (C)	1.89	0.2063	39.72	0.0002	428.48	0.0000
Nitrogen dose (N)	17.19	0.0032	71.66	0.0000	22.48	0.0000
Interaction (CxN)	0.07	0.8004	2.36	0.1630	0.25	0.6298

F-value of Fisher-Snedeco test, *p*—probability < 0.05.

**Table 2:** Estimated values of protein and gluten content (%) and gluten index depending on main factors: winter wheat lines and nitrogen fertilization doses. The second column shows the average values of the parameters listed in the first column, obtained for each of the wheat lines, and for both N doses (0 and 120 kg N·ha^−1^). The third column shows the average values of the parameters listed in the first column, obtained for each N doses, and for both tested wheat lines (wasko.gl+ and wasko.gl−).

Characteristics	Winter Wheat Line (The Average Value for Both Nitrogen Fertilization Doses)		Nitrogen Fertilization (kg N·ha^−1^) (The Average Value for Both Winter Wheat Lines)	
	wasko.gl−	wasko.gl+	N0	N120
Protein content (%)	14.3 ± 0.6 a	13.7 ± 0.5 b	12.9 ± 0.4 b	14.9 ± 0.9 a
Gluten content (%)	32.3 ± 2.5 b	39.1 ± 1.7 a	31.2 ± 0.7 b	40.2 ± 0.7 a
Gluten index	93.0 ± 3.0 a	48.3 ± 2.2 b	75.7 ± 0.3 a	65.4 ± 0.9 b

Within each row, values with the same letter are not significantly different (*p* < 0.05).

**Table 3:** Conformations of S-S bridges [%] calculated from FT-Raman spectra of wheat flour of wasko.gl+ and wasko.gl− lines treated with N fertilizers in doses: 0 (N0) and 120 (N120) kg N·ha^−1^ respectively. The wavenumbers of the bands’ positions (cm^−^^1^), obtained by curve-fitting spectra, are shown in the brackets. N ≥ 3, SD < 5%.

**Wheat Lines**	**SS_g-g-g_ [%]**	**SS_t-g-g_ [%]**	**SS_t-g-t_ [%]**
wasko.gl−, N0	3 (513)	54 (519, 523)	43 (529, 534)
wasko.gl−, N120	16 (517)	38 (523)	46 (529, 537)
wasko.gl+, N0	5 (514)	47 (520, 524)	48 (529, 536)
wasko.gl+, N120	-	35 (522)	65 (529, 539)

**Table 4:** The estimated content of the secondary structure of gliadin proteins isolated from flour obtained from kernels of wasko.gl− and wasko.gl+ wheat lines under minimal (N0) and maximal (N120) dose of nitrogen fertilization. N ≥ 3, SD < 5%.

	wasko.gl− (N0)	wasko.gl− (N120)	wasko.gl+ (N0)	wasko.gl+ (N120)
β–sheet (1627–33 cm^−1^)	19	20	23	20
α-helix (1648–50 cm^−1^)	29	30	31	25
RC (1663–1666 cm^−1^)	30	26	24	28
β-turn (1680–83 cm^−1^)	18	20	16	25
aβ-sheet (1691–98 cm^−1^)	4	4	6	2

## Data Availability

All data generated or analysed during this study are included in this published article. Moreover, the datasets used and/or analysed during the current study are available from the corresponding author on reasonable request.

## Supplementary Materials

The following supporting information can be downloaded at: https://www.mdpi.com/article/10.3390/molecules27175684/s1, Figure S1: The decomposition of the 500–550 cm^−1^ region obtained from the isolated gliadin proteins of wasko.gl+, panel (A), and wasko.gl−, panel (B) under nitrogen fertilization in doses 0 (N0) and 120 (N120) kg·ha^−1^. The experimental profiles are represented by dashed black lines and the calculated ones are represented by solid red lines. The calculated profiles in the panels were determined as the sums of the individual curve-fitted components typical for gauche–gauche–gauche (SS_g-g-g_) at 513-519 cm^−1^—blue line; for trans–gauche–gauche (SS_t-g-g_) in the range 519–524 cm^−1^—green line; and in the range 529–539 cm^−1^ for trans–gauche–trans (SS_t-g-t_) conformations—yellow line, respectively; Table S1: The most characteristic Raman bands obtained for the wheat kernels of wasko.gl+ and wasko.gl− lines treated with various doses of nitrogen fertilizers (N0 and N120); Table S2: The observed changes in relative intensities of gliadin bands of wasko.gl+ and wasko.gl− lines after nitrogen fertilization. The references [69,70,71,72,73,74,75,76,77,78] cited in Supplementary materials.

## Sample Availability

Samples of the compounds are not available from the authors.
