# Supplementary material for: Does Nitrogen Fertilization Affect the Secondary Structures of Gliadin Proteins in Hypoallergenic Wheat?
Source: Molecules. 2022 Sep 3;27(17):5684. doi: 10.3390/molecules27175684 (PMC9457604; doi:10.3390/molecules27175684)
Supplement: Supplementary file 1 [file molecules-27-05684-s001.zip › molecules-1858144-supplementary.pdf]

Supplementary Materials

Table S1. The most characteristic Raman bands obtained for the wheat kernels of wasko.gl+ and wasko.gl– lines treated with various doses of nitrogen fertilizers (N0 and N120).

Peak Number	Wavenumber/cm^{-1}	Chemical Components	References
1	440	polysaccharides	[25,69]
2	480	polysaccharides (marker band to identify presence of starch)	[25,70–72]
	500-550	S-S bridges	[36]
	640-670<br>700-745	C-S stretching vibrations of Met and Cys	[36]
	760	Trp	[73,74]
	835, 855	Tyr	[37]
3	867	polysaccharides (stretching or deformation modes)	[70-72]
4	938	polysaccharides (marker band for glycosidic linkage in starch)	[25,26,70,72]
5	1050	polysaccharides	[25,26,70,72]
6	1082	polysaccharides	[70]
7	1125	polysaccharides	[25,70,72]
8,9	1261, 1336	polysaccharides, lipids, fatty acids	[25,26,70,72]
10	1378	polysaccharides	[25,26,70]
11	1458	polysaccharides, lipids, fatty acids	[25,26,70,75,76]
12	1600	polyphenols, flavonoids	[77,78]
13	1657	amide I	[38,61]

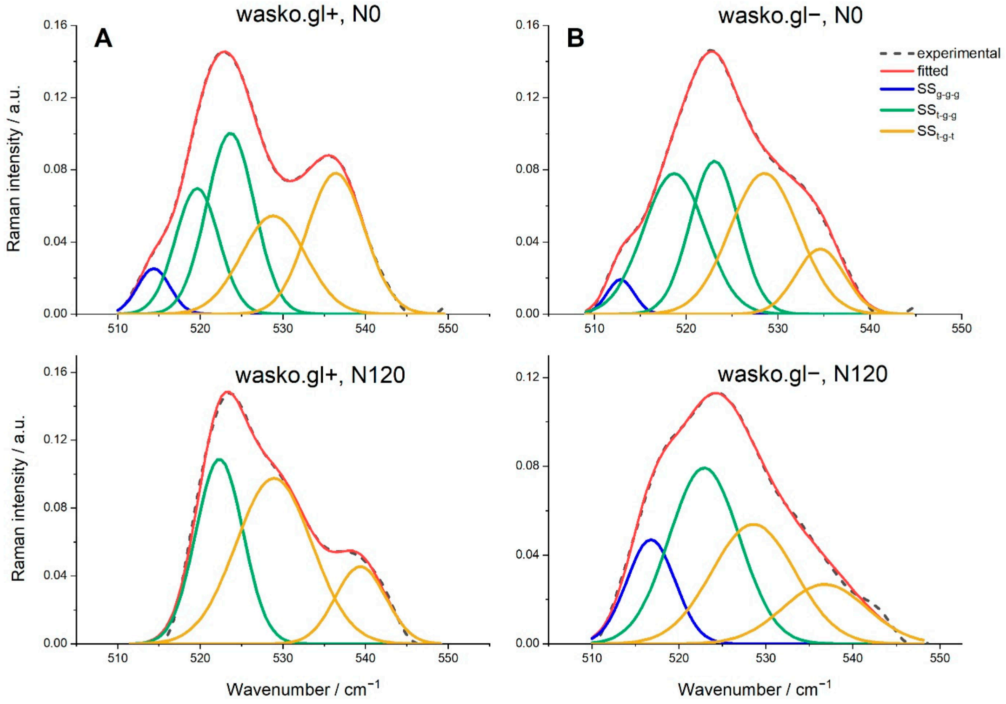

Figure S1. The decomposition of the 500–550 cm^{-1} region obtained from the isolated gliadin proteins of wasko.gl+, panel (A), and wasko.gl–, panel (B) under nitrogen fertilization in doses 0 (N0) and 120 (N120) kg·ha^{-1}. The experimental profiles are represented by dashed black lines and the calculated ones are represented by solid red lines. The calculated profiles in the panels were determined as the sums of the individual curve-fitted components typical for gauche–gauche–gauche ($SS_{g\text{-}g\text{-}g}$) at 513-519 cm^{-1}—blue line; for trans–gauche–gauche ($SS_{t\text{-}g\text{-}g}$) in the range 519–524 cm^{-1}—green line; and in the range 529–539 cm^{-1} for trans–gauche–trans ($SS_{t\text{-}g\text{-}t}$) conformations—yellow line, respectively.

Table S2. The observed changes in relative intensities of gliadin bands of wasko.gl+ and wasko.gl– lines after nitrogen fertilization.

Gliadin fraction	Band number	Relative change after N fertilization (%)†		Gliadin fraction	Band number	Relative change after N fertilization (%)		Gliadin fraction	Band number	Relative change after N fertilization (%)	
		wasko.gl-	wasko.gl+			wasko.gl-	wasko.gl+			wasko.gl-	wasko.gl+
	01	-9	-6		31	+41	+46		47	+12	-2
	02	-4	+49		32	+27	+34		48	-17	+5
	03	+6	+42		33	+26	+32		49	+22	-17
	04	-45	+53		34	+74	+80		50	n.d.	+78
	05	+10	+19		35	+24	+27		51	+26	-29
	06	-31	n.d.‡		36	+13	+14		52	+32	+64
	07	n.d.	+41`		37	-61	+36		53	+8	+68
	08	+17	+25		38	+52	+76		54	n.d.	n.d.
	09	+6	+33	α+β	39	+28	+29	γ	55	-4	+200
	10	-4	+17		40	+43	+48		56	-10	+41
	11	-4	+3		41	+94	+83		57	+18	+3
	12	+4	+5		42	+10	+16		58	+46	+25
	13	+3	-18		43	-43	-28		59	+34	+36
	14	+50	+25		44	+7	+17		60	+9	+231
	15	+15	+33		45	n.d.	n.d.		61	-4	+44
ω	16	+11	0		46	-41	+149		62	+26	+18
	17	+90	-1						63	n.d.	n.d.
	18	+1	+63						64	-5	+59
	19	-4	+7						65	+34	-40
	20	-43	+51						66	+14	-200
	21	+77	+100						67	n.d.	+98
	22	+27	+67						68	+15	-16
	23	+4	+49						69	n.d.	+171
	24	-2	+5						70	n.d.	-100
	25	+28	-1						71	-45	-100
	26	+69	+22								
	27	+54	-16								
	28	+2	+5								
	29	+7	+41								
	30	+46	+34								

†Relative change = 100×(N120 band$_i$ area- N0 band$_i$ area)/N0 band$_i$ area; i = 1, 2…71. ‡n.d. – band not detected
